# Supplementary material for: Objective Patterns of Face Recognition Deficits in 165 Adults with Self-Reported Developmental Prosopagnosia
Source: Brain Sci. 2019 Jun 6;9(6):133. doi: 10.3390/brainsci9060133 (PMC6627939; doi:10.3390/brainsci9060133)
Supplement: Supplementary file 1 [file brainsci-09-00133-s001.zip › brainsci-515298-supple/brainsci-515298-supple 2.pdf]

Supplementary Materials S2. SDT results for cases with significant dissociations between impaired famous face test (FFT) performance and intact CFPT and CFMT scores. The sequential Bonferroni correction is applied to correct for multiple comparisons.

Case	% Correct			FFT v CFPT			FFT v CFMT		
	**CFPT**	**CFMT**	**FFT**	*t*	*p*	**% More Extreme**	*T*	*p*	**% More Extreme**
DP001	73.61	72.22	30.95	4.18	**.001**	0.01	4.67	**.001**	0.01
DP002	87.50	73.61	55.10	3.55	**.001**	0.04	2.68	**.01**	0.48
DP013	59.72	70.83	36.00	2.69	**.01**	0.46	4.13	**.001**	0.01
DP016	77.78	62.50	16.95	5.54	**.001**	0.01	5.18	**.001**	0.01
DP018	79.17	73.61	55.56	2.84	**.01**	0.31	2.64	**.01**	0.53
DP020	73.61	58.33	45.00	3.16	**.01**	0.12	2.44	**.02**	0.89
DP024	65.28	80.56	15.91	4.61	**.001**	0.01	6.59	**.001**	0.01
DP025	77.78	66.67	27.91	4.74	**.001**	0.01	4.53	**.001**	0.01
DP029	81.94	59.72	46.15	3.75	**.001**	0.02	2.44	**.02**	0.89
DP033	76.39	81.94	38.81	5.75	**.001**	0.01	8.48	**.001**	0.01
DP034	79.17	70.83	60.00	3.79	**.001**	0.02	4.10	**.001**	0.01
DP037	62.50	75.00	52.00	2.99	**.01**	0.23	5.77	**.001**	0.01
DP040	69.44	59.72	28.30	6.18	**.001**	0.01	8.03	**.001**	0.01
DP045	72.22	81.94	68.97	2.15	**.04**	1.87	3.74	**.001**	0.03
DP048	80.56	91.67	57.14	4.23	**.001**	0.01	6.54	**.001**	0.01
DP058	63.89	72.22	61.67	2.10	**.04**	2.09	3.97	**.001**	0.01
DP060	68.06	66.67	28.81	5.99	**.001**	0.01	8.60	**.001**	0.01
DP061	72.22	66.67	42.37	4.97	**.001**	0.01	6.49	**.001**	0.01
DP064	69.44	70.83	60.00	4.10	**.01**	0.01	4.10	**.001**	0.01
DP065	68.06	61.11	53.33	3.40	**.01**	0.07	4.23	**.001**	0.01
DP077	66.67	69.44	54.24	3.16	**.01**	0.14	4.88	**.001**	0.01
DP080	65.28	69.44	50.91	3.38	**.01**	0.08	5.41	**.001**	0.01
DP087	63.89	68.06	44.19	3.82	**.001**	0.02	3.43	**.001**	0.07
DP090	68.06	56.94	52.83	3.36	**.01**	0.08	2.03	**.05**	2.44
DP091	76.39	54.17	40.00	5.45	**.001**	0.01	3.00	**.01**	0.23
DP094	70.83	73.61	29.17	5.99	**.001**	0.01	5.07	**.001**	0.01
DP095	66.67	58.33	37.74	4.74	**.001**	0.01	3.43	**.001**	0.07
DP098	76.39	69.44	34.88	5.96	**.001**	0.01	4.33	**.001**	0.01
DP100	61.11	63.89	35.59	4.42	**.001**	0.01	3.94	**.001**	0.01
DP104	69.44	66.67	56.76	3.10	**.01**	1.71	2.25	**.03**	1.49
DP105	81.94	66.67	47.46	5.24	**.001**	0.01	3.07	**.01**	0.19
DP113	59.72	56.94	41.38	3.70	**.001**	0.03	3.03	**.01**	0.21
DP125	55.56	73.61	38.60	3.58	**.001**	0.04	4.24	**.001**	0.01
DP128	65.28	58.33	48.33	3.54	**.001**	0.05	2.51	**.02**	0.81
DP129	65.28	58.33	28.33	5.54	**.001**	0.01	4.26	**.001**	0.01
DP130	65.28	55.56	19.28	6.44	**.001**	0.01	4.89	**.001**	0.01
DP139	56.94	51.39	27.27	3.92	**.001**	0.01	4.38	**.001**	0.01
DP142	75.00	66.67	48.72	4.02	**.001**	0.01	3.37	**.001**	0.07
DP154	59.72	52.78	34.62	3.58	**.001**	0.04	3.78	**.001**	0.02
DP157	56.94	59.72	40.00	2.80	**.01**	0.36	3.73	**.001**	0.02
DP158	58.33	58.33	22.92	2.42	**.02**	1.02	2.95	**.01**	0.27
DP165	47.22	41.67	8.33	2.42	**.02**	1.01	2.77	**.01**	0.43
